# Warburg effect and lactylation in cancer: mechanisms for chemoresistance

**DOI:** 10.1186/s10020-025-01205-6

**Published:** 2025-04-22

**Authors:** Wenjie Zhang, Min Xia, Jiahui Li, Gaohua Liu, Yan Sun, Xisha Chen, Jing Zhong

**Affiliations:** 1 Clinical Medical Research Center, The First Affiliated Hospital, Hengyang Medical School, University of South China, Hengyang, 421001 Hunan China; 2 Institute of Cancer Research, The First Affiliated Hospital, Hengyang Medical School, University of South China, Hengyang, 421001 Hunan China

**Keywords:** Cancer, Chemoresistance, Cancer therapy, Glycolysis, Lactylation

## Abstract

In the clinical management of cancers, the emergence of chemoresistance represents a profound and imperative “pain point” that requires immediate attention. Understanding the mechanisms of chemoresistance is essential for developing effective therapeutic strategies. Importantly, existing studies have demonstrated that glucose metabolic reprogramming, commonly referred to as the Warburg effect or aerobic glycolysis, is a major contributor to chemoresistance. Additionally, lactate, a byproduct of aerobic glycolysis, functions as a signaling molecule that supports lysine lactylation modification of proteins, which also plays a critical role in chemoresistance. However, it is insufficient to discuss the role of glycolysis or lactylation in chemoresistance from a single perspective. The intricate relationship between aerobic glycolysis and lactylation plays a crucial role in promoting chemoresistance. Thus, a thorough elucidation of the mechanisms underlying chemoresistance mediated by aerobic glycolysis and lactylation is essential. This review provides a comprehensive overview of these mechanisms and further outlines that glycolysis and lactylation exert synergistic effects, promoting the development of chemoresistance and creating a positive feedback loop that continues to mediate this resistance. The close link between aerobic glycolysis and lactylation suggests that the application of glycolysis-related drugs or inhibitors in cancer therapy may represent a promising anticancer strategy. Furthermore, the targeted application of lactylation, either alone or in combination with other treatments, may offer new therapeutic avenues for overcoming chemoresistance.

## Introduction

The underlying reasons for the occurrence and progression of cancer are abnormal signaling and metabolic reprogramming. Otto Warburg first postulated the Warburg effect, also known as aerobic glycolysis (hereafter referred to as glycolysis), in 1920. This phenomenon represents the most significant and classical phenotype in the metabolic reprogramming of cancer cells (Liao et al. 2024). Unlike normal cells, which supply energy through the catabolism of glucose via mitochondrial oxidative phosphorylation, tumor cells preferentially convert glucose to lactate and tend to rely on the glycolysis pathway for energy production, even in the presence of sufficient oxygen (Koppenol et al. 2011). This process provides essential biomolecules and raw materials for the survival of tumor cell microenvironments. Despite the complexity of cancer metabolism, glycolysis remains a key process (Paul et al. 2022).

The elevated uptake of glucose is typically accompanied by a high glycolytic flux, resulting in substantial lactate production (Ganapathy-Kanniappan and Geschwind 2013). As a metabolite produced at the end of the glycolysis process, lactate has also been demonstrated to act as a precursor substance mediating the modification of histone lysine known as histone lactylation, which was first described in 2019 (Zhang et al. 2019a). The synthesis of endogenous lactate determines the extent of histone lysine H3 K18 lactylation (Rho et al. 2023). Histone lactylation occurs at various lysine residues, including H3 K9, H3 K23, and H4 K12, and has been shown to effectively regulate cell signaling and gene expression (De Leo et al. 2024; Huang et al. 2023). Notably, lactylation is not restricted to histones; it has also been identified on various non-histone proteins, which have been proven to play a significant role in tumors. These new insights into lactylation modification of protein in tumor research indicate a novel direction in the study of tumorigenesis and development (Lv et al. 2023).

In the context of clinical care, the emergence of resistance to cancer chemotherapy drugs has been recognized as a significant factor contributing to tumor treatment failure. Consequently, the development of drug resistance has become a major challenge in tumor treatment. Available research indicates that DNA damage repair, epigenetic changes, immune evasion, and survival pathway activation are the primary causes of chemoresistance, all linked to glycolysis and lactylation (Li et al. 2020; Sun et al. 2024; Duan et al. 2024; Zhu et al. 2025; Chaudagar et al. 2023).

Elevated levels of lactate serve as the primary driving force behind the lactylation of proteins. The accumulation of lactate is a requisite condition for the occurrence of lactylation modifications. This indicates that any factor influencing glycolysis—particularly those that affect the expression and enzymatic activity of glycolytic enzymes—may modulate lactylation levels. While existing studies have examined the relationship between elevated lactate levels and lactylation, an epigenetic modification induced by lactate, as well as the correlation between lactylation and tumor drug resistance, a more comprehensive scientific perspective linking the reprogramming of glycolysis to lactylation remains lacking. Such a perspective is essential for facilitating a deeper exploration of the specific mechanisms underlying glycolysis and lactylation in the context of cancer chemotherapy resistance.

The close association between tumor glycolysis, lactylation, and chemoresistance has sparked interest in cancer glycolysis metabolism and has prompted sustained efforts to develop inhibitors targeting the glycolysis pathway or specific glycolytic enzymes. Emerging therapeutic strategies that focus on the lactylation of cancer proteins have begun to take shape. Through ongoing exploration and development, we have come to recognize that co-targeting glycolysis and lactylation may represent a novel and promising strategy for overcoming cancer chemoresistance.

This review summarizes the regulatory networks through which glycolysis and lactylation modifications of protein have mediated cancer chemoresistance in recent years, along with the associated metabolic inhibitors aimed at overcoming this resistance. The close relationship between glycolysis and lactylation is discussed, presenting a holistic perspective on how glycolysis and lactylation exert synergistic effects to mediate cancer chemoresistance. This provides an important theoretical basis for the development of new combination therapy strategies.

## Glycolysis and cancer chemoresistance

**Fig. 1:**
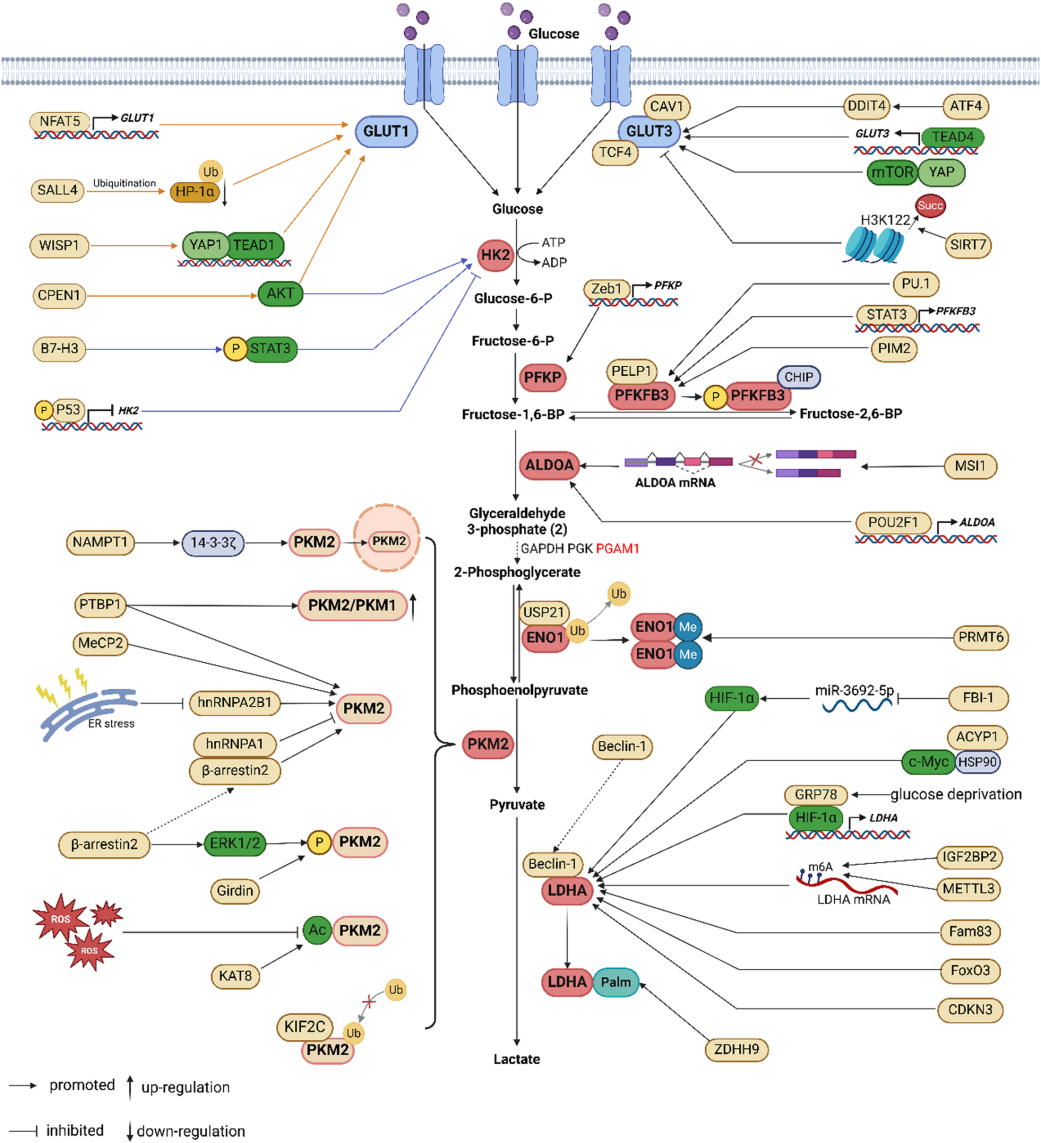
The regulatory network of glycolytic factors in cancer chemoresistance. These proteins regulate the expression and activity of glycolytic enzymes that facilitate glycolysis in tumors, ultimately contributing to the development of chemoresistance. The mechanisms they regulate include functioning as transcription factors, mediating RNA alternative splicing, inducing post-transcriptional and post-translational modifications, and activating classical signaling pathways et al

**Fig. 2:**
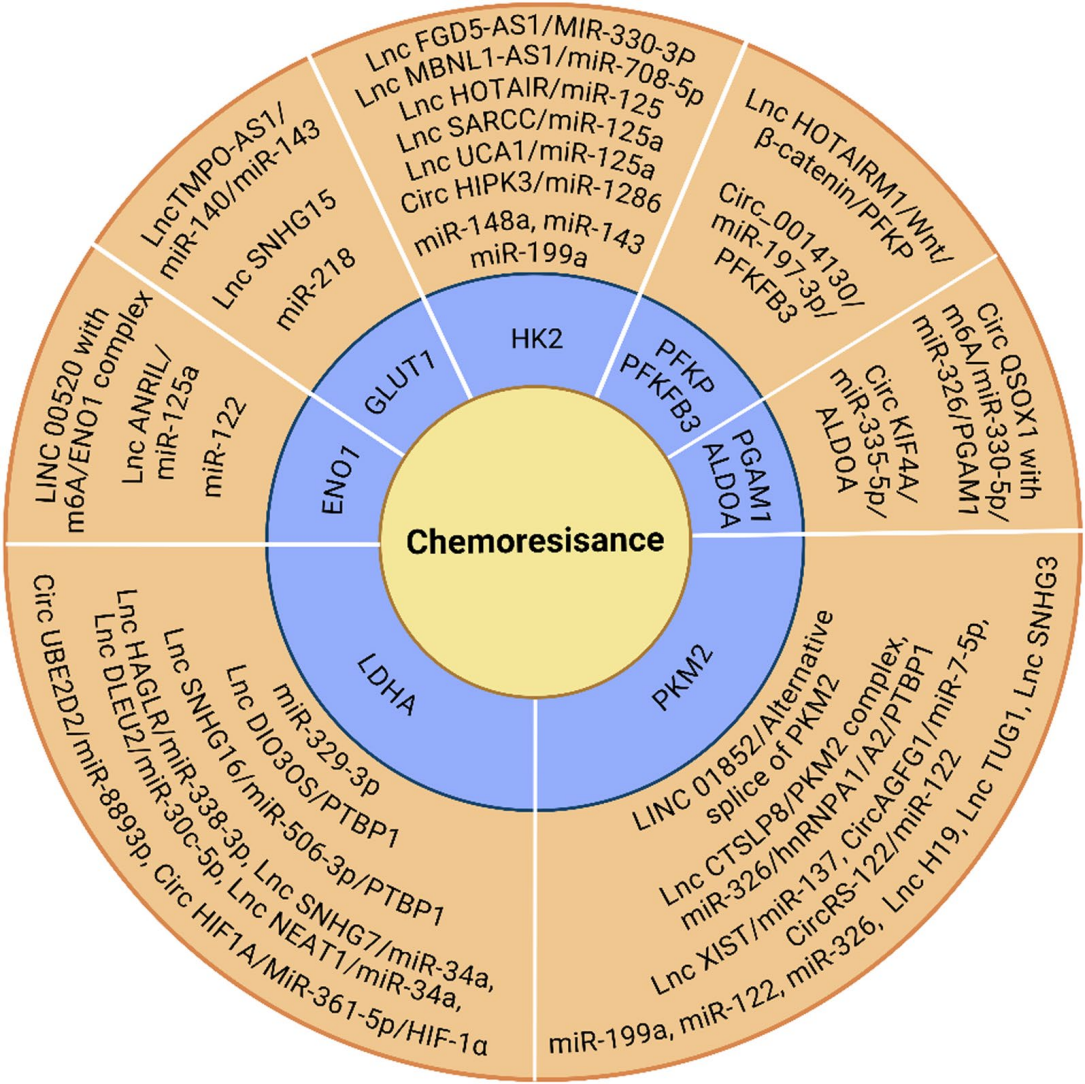
Non-coding RNAs regulate chemoresistance of cancer. NcRNAs can act as oncogenes or tumor suppressors, leading to resistance to cancer chemotherapy by directly targeting or forming networks of ceRNAs that mediate the epigenetic regulation of glycolytic factors

Since tumor cell-specific glycolysis is a crucial factor contributing to drug resistance (Icard et al. 2018), the abnormal dysregulation of glycolytic genes and glucose transporter proteins that induces chemoresistance is particularly noteworthy. A complex regulatory network involving glucose transporter proteins and glycolytic enzymes enhances resistance to cancer chemotherapy (see Fig. 1 and Table 1). Additionally, non-coding RNAs play a significant role in this regulatory network (see Fig. 2).

**Table 1:** The regulatory network of glycolytic enzymes in cancer chemoresistance

Gene	Expression	Downstream target	Drug resistance	Cancer	References
SALL4	Up	Ubiquitination of HP-1α/GLUT1	DOX	HCC	Kim et al. (2017)
NFAT5	Up	GLUT1	GEM	ICC	Gao et al. (2023)
CPEN1	Up	AKT/GLUT1	L-OHP	CRC	Wang et al. (2021a)
WISP1	Up	YAP1/TEAD1/GLUT1	DDP	LSCC	Wang et al. (2019a)
MiR-218	Down	GLUT1	DDP	Bladder cancer	Li et al. (2017a)
Lnc SNHG15	Up	GLUT1	5-FU	CRC	Li et al. (2023)
ATF4	Up	DDIT4/GLUT3	TMZ	GBM	Ho et al. (2020)
YAP	Up	mTOR/GLUT3	5-FU	CRC	Xu et al. (2024a)
		GLUT3/EGRF	TKI	AML	Zhuang et al. (2018)
		CAV-1/GLUT3 complex	TKI	NSCLC	Ali et al. (2019)
TGF4	Up	GLUT3 promoter	Vemurafenib	Melanoma	Liu et al. (2020)
		TEAD1/GLUT3 complex	PTX	Breast cancer	Li et al. (2022a)
SIRT7	Up	desuccinylation of GLUT3 H3 K122	GEM	Pancreatic cancer	Chen et al. (2024a)
LncTMPO-AS1	Up	miR-140/miR-143/GLUT1	PTX	EC	Dong et al. (2022)
B7-H3	Up	STAT3/HK2	L-OHP	CRC	Shi et al. (2019)
P53	Down	HK2	DDP	Ovarian cancer	Han et al. (2019)
MiR-148a	Down	HK2	DDP	Cervical cancer	Yang et al. (2021)
MiR-143	Down	HK2	5-FU	CRC	Chen et al. (2023)
MiR-199a	Down	HK2	Sorafenib	HCC	Zhou et al. (2023)
Lnc FGD5-AS1	Up	miR-330-3p/HK2	5-FU	CRC	Gao et al. (2021)
Lnc MBNL1-AS1	Up	miR-708-5p/HK2	Tripterine	HCC	Zhang et al. (2024)
Lnc HOTAIR	Up	miR-125/HK2	TMZ	GBM	Zhang et al. (2020)
Lnc SARCC	Up	miR-125a/HK2	CDDP	Osteosarcoma	Wen et al. (2020)
Lnc UCA1	Up	miR-125a/HK2	ADR	AML	Zhang et al. (2018)
Circ HIPK3	Up	miR-1286/HK2	PTX	Breast cancer	Ni et al. (2021)
Zeb1	Up	PFKP	EPI, ETOP	Breast cancer	Jiang et al. (2022a)
Lnc HOTAIRM1	Up	Wnt/β-catenin/PFKP	Ara-c	AML	Chen et al. (2020a)
PU.1	Up	PFKFB3	TKIs	CML	Zhu et al. (2018)
STAT3	Up	PFKFB3	TKIs	Lymphoma	Hu et al. (2022a)
		PELP1/PFKFB3 complex	PTX	Breast cancer	Truong et al. (2021)
PIM2	Up	P-PFKFB3	PTX	Breast cancer	Lu et al. (2021)
Circ_0014130	Up	miR-197-3p/PFKFB3	5-FU	CRC	Wang et al. (2022)
POU2 F1	Up	ALDOA	L-OHP	CRC	Lin et al. (2022)
MSI1	Up	ALDOA, the mTOR pathway	TAM	Breast cancer	Yu et al. (2024)
Circ KIF4 A	Up	miR-335-5p/ALDOA	TMZ	Glioma	Luo et al. (2022)
Circ QSOX1 with m6 A	Up	miR-330-5p/miR-326/PGAM1	D9D	CRC	Liu et al. (2022)
USP21	Up	ENO1	GEM	Cholangiocarcinoma	Xu et al. (2024c)
PRMT6	Up	Methylation of ENO1	DDP	Lung cancer	Sun et al. (2023a)
LINC 00520 with m6 A	Up	LINC 00520/ENO1 complex/FBXW7	DDP	Osteosarcoma	Wei et al. (2024)
MiR-122	Down	ENO1	DDP	Gastric cancer	Qian et al. (2017)
Lnc ANRIL	Up	miR-125a/ENO1	ADR	TNBC	Ma et al. (2022)
PSMD10	Up	GLUT1/HK2 and PKM2		HCC	Liu et al. (2019)
PTEN	Up	AKT/GLUT1 and HK2	Idarubicin, Ara-c	AML	Ryu et al. (2019)
NgBR	Up	HIF-1α/GLUT1, HK2, and LDHA	PTX	Breast cancer	Liu et al. (2023)
miR-149-3p	Down	GLUT1, HK2 and LDHA	ADR, DDP	AML	Chen et al. (2024 d)
Lnc UCA1	Up	HK2 and LDHA	PTX	CRC	Shi et al. (2021)

### GLUT1 and GLUT3

Glucose uptake mediated by the facilitative glucose transporters (GLUTs) serves as the foundational step in cellular glucose metabolism and represents the rate-limiting factor in this process (Guerrero et al. 2024). The GLUTs, which are encoded by the SLC2 A gene, are dysregulated in various types of cancer (Macheda et al. 2005; Wu et al. 2020; Hao et al. 2024; Nagarajan et al. 2017; Barbosa and Martel 2020). For example, GLUT1 and GLUT3 are upregulated in breast cancer (Krzeslak et al. 2012), CRC (GabAllah et al. 2017; Dai et al. 2020), and non-small cell lung cancer (NSCLC) (Younes et al. 1997), and are closely associated with cancer progression. Furthermore, the aberrant expression of GLUT1 and GLUT3 is a critical factor contributing to chemoresistance in tumors (Ma and Zong 2020).

The dysregulation of certain oncogenes or tumor suppressor genes can promote GLUT1-induced glycolysis, thereby accelerating chemoresistance. In hepatocellular carcinoma (HCC) cells, the expression of GLUT1 is upregulated by spalt-like transcription factor 4 (SALL4) in a heterochromatin protein 1α (HP1α)-dependent manner, which confers doxorubicin (DOX) resistance to HCC cells by enhancing DNA damage responses (Kim et al. 2017). Additionally, in intrahepatic cholangiocarcinoma (ICC), the upregulation of nuclear factor of activated T cells 5 (NFAT5) increases GLUT1 expression and induces gemcitabine (GEM) resistance (Gao et al. 2023). Furthermore, the chemoresistance induced by GLUTs is associated with several carcinogenic signaling pathways, including Akt and Wnt1 (Wang et al. 2021a; Wang et al. 2019a).

Numerous studies have indicated that the non-coding RNA (ncRNA) regulatory network facilitates cancer chemoresistance by enhancing GLUT1-related glycolysis. The down-regulation of microRNA-218 (miR-218) increases GLUT1 expression, thereby conferring cisplatin (DDP) resistance in bladder cancer (Li et al. 2017a). In colorectal cancer (CRC) cells, long noncoding RNA small nucleolar RNA host gene 15 (lncRNA SNHG15) up-regulates GLUT1 expression, leading to 5-fluorouracil (5-FU) resistance (Li et al. 2023). Additionally, in endometrial cancer cells, GLUT1 induces paclitaxel (PTX) resistance through lncRNA thymopoietin-antisense RNA-1 (TMPO-AS1) (Dong et al. 2022). Furthermore, the phenomenon of cancer chemoresistance, characterized by the aberrant expression of GLUT1, is also evident in DDP resistance of ovarian cancer and the acquired imatinib resistance of gastrointestinal stromal tumor cells (Loar et al. 2010; Shima et al. 2022).

Similar to GLUT1, GLUT3 exhibits a higher glucose affinity than GLUT2 and GLUT4 (Simpson et al. 2008). This characteristic of high glucose turnover enables GLUT3 to play a crucial role in chemoresistance.

In glioblastoma (GBM), the overexpression of GLUT3 activates glycolysis that contributes to resistance against bevacizumab (Kuang et al. 2017). Similarly, treatment with temozolomide (TMZ) induces the overexpression of activating transcription factor 4 (ATF4), which promotes DNA damage-inducible transcript 4 (DDIT4)/GLUT3 signaling and enhances resistance to TMZ in GBM (Ho et al. 2020). Furthermore, it has been verified that the YAP/mTOR/GLUT3 pathway contributes to 5-FU resistance in CRC (Xu et al. 2024a). In acute myeloid leukemia (AML), GLUT3 negatively regulates epidermal growth factor receptor (EGFR) activity and its downstream signaling pathways, contributing to tyrosine kinase inhibitors (TKIs) chemoresistance (Zhuang et al. 2018).

Moreover, Ali et al. (2019) discovered that in TKIs-resistant NSCLC cells, caveolin-1 (CAV1) interacts with GLUT3, facilitating high glucose uptake that sustains the survival of resistant cells. In melanoma, transcription factor 4 (TCF4) binds to GLUT3 and enhances its expression, leading to resistance against vemurafenib (Liu et al. 2020). Similarly, in breast cancer, TEA domain transcription factor 4 (TEAD4) binds to the GLUT3 promoter, increasing its expression and thereby contributing to PTX chemoresistance (Li et al. 2022a). Post-translational modifications (PTMs) of GLUT3 also play a significant role in cancer chemoresistance. Sirtuin 7 (SIRT7) inhibits the expression of GLUT3 by desuccinylating its H3 K122 residue, which subsequently decreases GEM uptake and confers resistance in pancreatic cancer (Chen et al. 2024a).

Notably, the secretion of Interleukin 3 (IL-3) in Yes-associated protein 1 (YAP1) overexpressing and 5-FU-resistant gastric cancer cells induces tumor-associated macrophages to undergo M2-type polarization, promoting their GLUT3 expression and activating glycolysis. The activated M2-type tumor-associated macrophages secrete increased levels of chemokine ligand 8 (CCL8), which ultimately induces 5-FU resistance in tumor cells via the JAK/STAT signaling pathway (He et al. 2021).

In addition to the dysregulation of GLUT expression affecting the reprogramming of glycolytic metabolism in tumor cells, which results in chemoresistance, the crosstalk between tumor cells and the tumor microenvironment must not be overlooked. This crosstalk forms a signaling network that offers new insights into the role of GLUTs in cancer drug resistance and presents innovative strategies for overcoming chemoresistance in cancer research. These findings collectively reveal that GLUTs, particularly GLUT1 and GLUT3, play a fundamental role in the development of drug resistance.

### HK2

The initial key enzymatic phase of glycolysis involves the conversion of glucose to glucose 6-phosphate (G-6-P) through the catalytic action of hexokinase (HK), a crucial rate-limiting enzyme. As the most active isoenzyme in the HK family, the activation of HK2 drives tumor metabolism toward glycolysis in various malignant tumors, thereby regulating tumor progression and chemoresistance (Ciscato et al. 2021; Xie et al. 2023).

In CRC, the immunoregulatory protein B7 homolog 3 (B7-H3) promotes the expression of HK2 through the phosphorylation of signal transducer and activator of transcription 3 (STAT3), thereby conferring resistance to 5-FU and oxaliplatin (L-OHP) (Shi et al. 2019). The Ser15 phosphorylated P53 inhibits the transcription of HK2 by binding to its promoter, thereby suppressing glycolysis and rendering ovarian cancer cells sensitive to DDP (Han et al. 2019).

NcRNAs play a crucial role in modulating HK2-induced glycolysis, thereby promoting tumor chemoresistance. Research conducted by Yang et al. (2021) demonstrated that the down-regulation of miR-148a enhances the expression of HK2, subsequently conferring cervical cancer resistance to DDP. In CRC, the attenuation of miR-143 up-regulates HK2 expression, thereby conferring 5-FU resistance (Chen et al. 2023). Long non-coding RNAs (LncRNAs) have been recognized for their role as competitive endogenous RNAs (ceRNAs). For instance, lncRNA FGD5-AS1 (RhoGEF and PH domain containing 5 antisense RNA 1) up-regulates HK2 expression by directly sponging miR-330-3p, thereby conferring 5-FU resistance in CRC (Gao et al. 2021). Similarly, in other cancers, lncRNA MBNL1-AS1 (muscle blind-like protein 1 antisense RNA 1) confers HCC resistance to tripterine through the lncRNA MEBNL-AS1/miR-708-5p/HK2 axis (Zhang et al. 2024). The lncRNA HOTAIR/miR-125/HK2 axis contributes to TMZ resistance in GBM (Zhang et al. 2020). Additionally, lncRNA-SARCC (lncRNA suppressing androgen receptor in renal cell carcinoma) and lncRNA UCA1 (urothelial carcinoma-associated 1) can both increase HK2 expression by suppressing miR-125a, thereby conferring DDP and Adriamycin (ADR) resistance, respectively, in osteosarcoma and AML (Wen et al. 2020; Zhang et al. 2018). Furthermore, Ni et al. (2021) identified a circular RNA (circRNA) named circRNA HIPK3 (homeodomain-interacting protein kinase 3) that promotes HK2 expression by sponging miR-1286, thereby conferring PTX resistance in breast cancer.

### PFKP and PFKFB3

As the subsequent crucial rate-limiting step, phosphofructokinase (PFK), particularly PFK-1, catalyzes the conversion of fructose 6-phosphate (F-6-P) to fructose 1,6-bisphosphate (F-1,6-BP). PFK-1 serves as the most important allosteric regulatory enzyme in glycolysis and exists in three distinct isoforms present in different tissues. Among these isoforms, the platelet-specific phosphofructokinase (PFKP) is highly expressed in various tumors (Shen et al. 2020; Chen et al. 2022a; Wang et al. 2013) and is considered a cancer-specific isoform. Furthermore, the enzymatic activity of PFK-1 is influenced by cytoplasmically localized metabolites, especially fructose-2,6-bisphosphate (F-2,6-BP), which is produced by 6-phosphofructo-2-kinase/fructose-2,6-bisphosphatase 3 (PFK-2/FBPase-2, PFKFB3) (Kotowski et al. 2021) and serves as the strongest allosteric agonist for PFK-1. Consequently, PFKFB3 plays a significant role in the regulatory network of glycolysis.

Cancer chemoresistance is associated with PFKP-mediated high glycolysis. For instance, the overexpression of PFKP confers resistance to 5-FU in CRC (Deng et al. 2024). Furthermore, zinc finger E-box binding homeobox 1 (Zeb1) directly enhances the transcription of PFKP, thereby contributing to epirubicin (EPI) and etoposide (ETOP) resistance in breast cancer (Jiang et al. 2022a). Similarly, PFKFB3-mediated high glycolysis also leads to chemoresistance. Yao et al. (2022) reported elevated glycolytic flux and upregulation of PFKFB3 in human epidermal growth factor receptor-2 (HER2) positive trastuzumab-resistant gastric cancer cells. In small-cell lung carcinoma (SCLC), high levels of PFKFB3 in cancer stem cells (CSCs) promote resistance to DOX, ETOP, and 5-FU (Thirusangu et al. 2022). Moreover, PFKFB3 expression is upregulated by transcription factors such as the Ets transcription factor (PU.1) and STAT3, which are associated with chemoresistance in cancers (Zhu et al. 2018; Hu et al. 2022a).

As the primary cause of cancer-related deaths in females, chemoresistance in breast cancer is often associated with the abnormal expression of PFKFB3, such as in tamoxifen (TAM) resistance (Zhao et al. 2024). In PTX-resistant breast cancer, proline, glutamic acid, leucine-rich protein 1 (PELP1) directly interacts with PFKFB3, leading to breast cancer cells exhibiting high glycolytic characteristics (Truong et al. 2021). Furthermore, proviral insertion in murine lymphomas 2 (PIM2) mediates the PFKFB3 Ser478 phosphorylation, which stabilizes the protein through the C-terminus of Hsc70-interacting protein (CHIP)-mediated ubiquitin–proteasome pathway (Lu et al. 2021). Additionally, PFKP and PFKFB3 are regulated by ncRNAs. Lnc RNA HOTAIRM1 (homeobox antisense intergenic RNA myeloid 1) promotes PFKP expression by activating the Wnt/β-catenin pathway, thereby conferring resistance to cytarabine (Ara-C) (Chen et al. 2020a). Wang et al. (2022) discovered that the upregulation of circ_0014130 enhances the expression of PFKFB3 by sponging miR-197-3p, which confers resistance to 5-FU in CRC cells. However, Xu et al. (2024b) provide an intriguing insight. Their research indicates that the inhibition of tyrosine phosphatase Src homology region 2 domain-containing phosphatase 1 (SHP-1) upregulates the PFKP expression via the Akt/β-catenin axis and reinforces glycolysis, breaking the metabolic quiescence of chemoresistance leukemia stem cells. Surprisingly, the enhanced glycolysis increases chemosensitivity to Ara-C, DOX, and azacitidine in AML. This finding is incongruous with previous reports, suggesting a dual role of glucose metabolic remodeling that requires further exploration.

### ALDOA

Fructose 1,6-bisphosphate is reversibly converted to glyceraldehyde 3-phosphate and dihydroxyacetone phosphate by aldolase (ALDOA, ALDOB, and ALDOC), thereby facilitating the progression of glycolysis. As a pivotal glycolytic enzyme, ALDOA is predominantly expressed in muscle and brain tissue, reprogramming glucose metabolism to support the malignant progression of tumors (Tang and Cui 2024). Furthermore, emerging evidence indicates a potential link between ALDOA-mediated glycolysis and chemotherapeutic resistance.

Aberrant expression of ALDOA has been shown to result in DDP resistance in lung cancer and 5-FU resistance in CRC (Kawai et al. 2017; Chang et al. 2020). Similarly, in CRC, the POU domain 2-like transcription factor 1 (POU2 F1) enhances ALDOA activity by directly binding to the ALDOA promoter, thereby conferring resistance to L-OHP (Lin et al. 2022). Furthermore, the regulation of ALDOA at the transcriptional level also affects cancer chemoresistance. Yu et al. (2024) demonstrated that Musashi RNA binding protein 1 (MSI1) mediates the inclusion of exon 7.2 in the 5′ untranslated region (5′ UTR) of ALDOA mRNA, which increases ALDOA expression, subsequently promoting the mammalian target of rapamycin (mTOR) pathway and conferring resistance to TAM in breast cancer.

The ceRNA regulatory network also plays a significant role. For instance, the overexpression of circKIF4 A (circular RNA kinesin family member 4 A) competitively interacts with miR-355-5p, leading to enhanced ALDOA expression and increased TMZ resistance in glioma (Luo et al. 2022).

### PGAM1

Phosphoglycerate mutase 1 (PGAM1), an isoform of phosphor-glycerate translocase, reversibly catalyzes the conversion of 3-phospho-glycerate to 2-phospho-glycerate during glycolysis. The overexpression of PGAM1 in various cancers has been shown to upregulate glycolysis, thereby promoting the survival of tumor cells (Sharif et al. 2019).

Aberrant expression of PGAM1 mediates high glycolytic flux and modulates ovarian cancer cells that are resistant to PTX (Feng et al. 2022). Liu et al. (2022) demonstrated that circQSOX1 (quiescin sulfhydryl oxidase 1) with the N6-methyladenosine (m6 A) modification is overexpressed and upregulates PGAM1 by sponging miR-330-5p and miR-326, thereby promoting glycolysis and lactate accumulation. The elevated levels of lactate can further support the proliferation of tumor-infiltrating regulatory T cells (Tregs) and enhance Treg-mediated anti-tumor immunity, thereby decreasing the sensitivity of CRC to anti-CTLA-4 therapy (D9D).

### ENO1

Alpha-enolase (ENO1), also designated as 2-phospho-D-glycerate hydrolase, is responsible for the transformation of 2-phosphoglycerate into phosphoenolpyruvate. Among the four isoforms of enolase, ENO1 accounts for the majority of cytosolic enolase activity in the glycolysis pathway (Muller et al. 2012). Furthermore, aberrant expression of ENO1 has been verified in several cancer types (Huang et al. 2022).

The classical role of ENO1 as a glycolytic enzyme is to influence glycolysis, and ENO1-mediated glycolysis has been demonstrated to confer chemoresistance. ENO1-related chemoresistance can be regulated by PTMs. For instance, ENO1 is directly stabilized by ubiquitin-specific protease 21 (USP21), which enhances chemoresistance to GEM in cholangiocarcinoma (Xu et al. 2024c). Additionally, protein arginine methyltransferase 6 (PRMT6) mediated methylation at R9 and R372 of ENO1 promotes the formation of its active form and facilitates the binding of 2-phospho-glycerate to ENO1, thereby upregulating glycolysis and DDP resistance in lung cancer (Sun et al. 2023a). The expression of ENO1, regulated by ncRNAs, is associated with glycolysis-mediated cancer chemoresistance. The m6 A modification of LINC00520 interacts with ENO1 and inhibits F-box and WD repeat domain containing 7 (FBXW7) mediated ENO1 ubiquitination and proteasomal degradation. This stabilization of the ENO1 protein ultimately increases the rate of glycolysis and contributes to DDP resistance in osteosarcoma (Wei et al. 2024). The silencing of miR-122 promotes the expression of ENO1, thereby conferring DDP resistance in gastric cancer (Qian et al. 2017). LncRNA ANRIL (antisense noncoding RNA in the INK4 locus) enhances ENO1 expression by downregulating miR-125a, thus boosting glycolysis and contributing to ADR resistance in triple-negative breast cancer (TNBC) (Ma et al. 2022). Gu et al. (2022) found that aberrant expression of ENO1 induces CRC cells to become resistant to 5-FU by activating the epithelial-mesenchymal transition (EMT) pathway, which may be related to glycolysis. Moreover, ENO1 mediates chemoresistance by regulating reactive oxygen species (ROS) homeostasis (Wang et al. 2019b), the PI3 K/AKT pathway, which reduces chemotherapy-induced apoptosis and cell cycle arrest (Chen et al. 2020b), and the Wnt signaling pathway (Mohapatra et al. 2021).

It is noteworthy that the non-enzymatic function of ENO1 has been observed. Sun et al. (2023b) revealed that ENO1 functions as an RNA-binding protein, accelerating the translation of YAP1 by binding to its mRNA, thereby mediating liver carcinogenesis. Additionally, Ma et al. (2024) found that ENO1 confers GEM resistance in pancreatic cancer through the Hippo signaling pathway by elevating YAP1 expression, suggesting that the moonlighting function of ENO1 similarly influences cancer chemoresistance.

### PKM2

Pyruvate kinase (PKM) is a key enzyme in the final stage of glycolysis, catalyzing the conversion of phosphoenolpyruvate (PEP) to pyruvate, while phosphorylating ADP to form ATP. The two isoforms encoded by the PKM gene are PKM1 and PKM2, with PKM2 being predominantly expressed in cancers (Zhu et al. 2021). It has been demonstrated that the kinase function of PKM2 regulates glycolysis and contributes to chemoresistance (Qin et al. 2022; Chan et al. 2023), which is summarized in Table 2.

**Table 2:** The regulatory network of PKM2 and LDHA in cancer chemoresistance

Gene	Expression	Downstream target	Drug resistance	Cancer	References
MeCP2	Up	PKM2	5-FU	Gastric cancer	Qin et al. (2022)
PTBP1	Up	Up-regulated PKM2/PKM1 ratio	TAM	Breast cancer	Gao et al. (2024)
	Up	PKM2	Vincristine L-OHP	CRC	Cheng et al. (2018)
NAMPT	Up	14–3-3ζ/PKM2	TAM	Breast cancer	Ge et al. (2019)
KIF2 C	Up	KIF2 C/PKM2 complex	DOX	Breast cancer	Jiang et al. (2021)
HnRNPA2B1	Down	PKM2	Sorafenib	HCC	Zhou et al. (2021)
HnRNPA1	Down	PKM2	Sorafenib	HCC	Zhang et al. (2019b)
	Up	Alternative splice of PKM2	DTX	Castration-resistant prostate cancer	Zhou et al. (2023)
PCAF	Up	Acetylation of PKM2	ETOP	Renal cell carcinoma	Shanmugasundaram et al. (2017)
KAT8	Up	Acetylation of PKM2	DDP	Lung cancer	Li et al. (2024a)
Girdin	Up	Phosphorylation of PKM2	DDP	Lung adenocarcinoma	Cao et al. (2022)
β-arrestin2	Up	Phosphorylation of PKM2	DTX	Castration-resistant prostate cancer	Zhou et al. (2023)
MiR-199a	Down	PKM2	Sorafenib	HCC	Li et al. (2018)
MiR-122	Down	PKM2	DTX	Prostatic cancer	Zhu et al. (2020)
Lnc H19	Down	PKM2	Erlotinib	Lung cancer	Chen et al. (2020c)
Lnc TUG1	Up	PKM2	ADR	AML	Chen et al. (2019)
Lnc SNHG3	Up	PKM2	Enzalutamide	castration-resistant prostate cancer	Yao et al. (2024)
Lnc CTSLP8	Up	Lnc CTSLP8/PKM2 complex	DDP	Ovarian cancer	Li et al. (2022b)
MiR-326	Down	PKM2	Sorafenib	CRC	Wu et al. (2022)
		hnRNPA1/A2/PTBP1/PKM2	Sorafenib	CRC	Wu et al. (2022)
Lnc XIST	Up	miR-137/PKM2	5-FU	CRC	Zheng et al. (2021)
LINC 01852	Down	Alternative splice of PKM2	5-FU, L-OHP	CRC	Bian et al. (2024)
CircAGFG1	Up	miR-7-5p/PKM2	L-OHP	CRC	Chen et al. (2024b)
CircRS-122	Up	miR-122/PKM2	L-OHP	CRC	Wang et al. (2020)
Beclin-1	Up	Beclin-1/LDHA complex	TAM	Breast cancer	Das et al. (2019)
FoxO3	Down	LDHA	TAM	Breast cancer	Fiorillo et al. (2023)
FBI-1	Up	miR-3692-5p/HIF-1α/LDHA	Sorafenib, Regorafenib, Lenvatinib, Cabozantinib	HCC	Liu et al. (2019)
ACYP1	Up	MYC/LDHA	LenvatinIb	HCC	Wang et al. (2023)
Fam83	Up	LDHA	GEM	Pancreatic cancer	Hua et al. (2021)
GRP78	Up	GRP78/HIF-1α/LDHA	GEM	Pancreatic cancer	Zhao et al. (2023)
METTL3	Up	LDHA	5-FU	CRC	Zhang et al. (2022)
IGF2BP2	Up	LDHA	5-FU	CRC	Jiang et al. (2022b)
ZDHH9	Up	Palmitoylation of LDHA	GEM	Pancreatic cancer	Chen et al. (2024c)
MiR-329-3p	Down	LDHA	DDP	Osteosarcoma	Li et al. (2021)
Lnc DIO3OS	Up	PTBP1/LDHA	Aromatase inhibitor	Breast cancer	Chen et al. (2022b)
Lnc SNHG16	Up	miR-506-3p/PTBP1/LDHA	5-FU	Gastric cancer	Ding et al. (2022)
Lnc HAGLR	Up	MiR-338-3p/LDHA	5-FU	Gastric cancer	Hu et al. (2022b)
Lnc SNHG7	Up	miR-34a/LDHA	DDP	Gastric cancer	Pei et al. (2021)
Lnc DLEU2	Up	MiR-30c-5p/LDHA	PTX	Gastric cancer	Xiang et al. (2024)
Lnc NEAT1	Up	MiR-34a/LDHA	5-FU	Cervical cancer	Shao et al. (2021)
Circ UBE2D2	Up	MiR-8893p/LDHA	Sorafenib	HCC	Huang et al. (2021)
Circ HIF1 A	Up	MiR-361-5p/HIF-1α/LDHA	Cetuximab	CRC	Geng et al. (2024)

The up-regulation of PKM2-induced chemoresistance frequently occurs in breast cancer, particularly in relation to TAM, DOX, and ADR resistance (Wang et al. 2021b; Yu et al. 2020; Qian et al. 2018). Gao et al. (2024) demonstrated that polypyrimidine tract-binding protein 1 (PTBP1) promotes glycolysis and enhances TAM resistance by increasing the ratio of PKM2 to PKM1. Moreover, the overexpression of nicotinamide phosphoribosyltransferase (NAMPT) mediates nuclear accumulation of PKM2, thereby enhancing TAM resistance through the up-regulation of 14‐3‐3ζ (Ge et al. 2019). Jiang et al. (2021) verified that kinesin family member 2 C (KIF2 C) interacts with and stabilizes PKM2, resulting in breast cancer resistance to DOX. Resistance to sorafenib also poses a challenge in HCC treatment, and research has demonstrated that aberrant expression of PKM2 significantly induces sorafenib resistance (Feng et al. 2020). Zhou et al. (2021) showed that endoplasmic reticulum (ER) stress up-regulates the expression of PKM2-related glycolysis by reducing the level of heterogeneous nuclear ribonucleoprotein A2/B1 (hnRNPA2B1), thereby promoting sorafenib resistance. Similarly, Zhang et al. (2019b) found that the attenuation of hnRNPA1 also up-regulates PKM2, conferring sorafenib resistance to HCC cells.

Different PTMs, such as acetylation and phosphorylation, serve as the primary mechanisms through which PKM2 exerts its roles in cancer. For instance, the stability of PKM2 is maintained through acetylation, which further contributes to cancer chemoresistance. Mechanistically, in renal cell carcinoma, the abundance of ROS inhibits the P300/CBP-associated factor (PCAF)-dependent acetylation of PKM2 the lysine residue 305 site (K305), contributing to ETOP resistance (Shanmugasundaram et al. 2017). Additionally, lysine acetyltransferase 8 (KAT8) acetylates PKM2 at K433, which is associated with DDP resistance in lung cancer (Li et al. 2024a). The Akt substrate Girdin promotes the phosphorylation of PKM2 the tyrosine residue 105 (Y105), thereby mediating DDP resistance in lung adenocarcinoma (Cao et al. 2022). Furthermore, PKM2 can also be phosphorylated by β-arrestin2 via the extracellular regulated protein kinases 1/2 (ERK1/2) signaling pathway or alternatively spliced by hnRNPA1, leading to a significant increase in the glycolysis rate and conferring docetaxel (DTX) resistance in castration-resistant prostate cancer (Zhou et al. 2023).

Numerous ncRNAs have been shown to target PKM2, contributing to cancer chemoresistance (see Fig. 2 and Table 2). The downregulation of miR-199a, miR-122, and lncRNA H19 (H19 imprinted maternally expressed transcript) increases PKM2 expression, thereby inducing resistance to sorafenib, DTX, and erlotinib, respectively (Li et al. 2018; Zhu et al. 2020; Chen et al. 2020c). Additionally, lncRNA TUG1 (Taurine-upregulated gene 1) upregulates PKM2 expression to mediate ADR resistance in AML (Chen et al. 2019). The lncRNA SNHG3 enhances PKM2 expression by inhibiting miR-139-5p, thereby increasing enzalutamide resistance in castration-resistant prostate cancer (Yao et al. 2024). Furthermore, lncRNA CTSLP8 (cathepsin L pseudogene 8) stimulates c-Myc expression by forming a transcription complex with PKM2, which enhances glycolysis and DDP resistance in ovarian cancer (Li et al. 2022b).

In CRC, the robust regulatory network of PKM2 enables cancer cells to exhibit increased resistance to chemotherapy. PTBP1 up-regulates the expression of PKM2, conferring resistance to vincristine and L-OHP (Cheng et al. 2018). The attenuation of miR-326 enhances the expression of PKM2, whether through direct up-regulation or indirectly via the miR-326-hnRNPA1/A2/PTBP1/PKM2 axis, contributing to sorafenib resistance (Wu et al. 2022). LncRNA XIST (X inactive specific transcript) impedes the conversion from PKM2 to PKM1 by inhibiting miR-137; thus, the lncRNA XIST/miR-137 axis confers 5-FU resistance through the up-regulation of the PKM2/PKM1 ratio (Zheng et al. 2021). LINC01852 increases the expression of PKM2 by inhibiting the alternative splicing of PKM, thereby sensitizing CRC cells to 5-FU and L-OHP (Bian et al. 2024). Similarly, circAGFG1 (ArfGAP with FG repeats 1) enhances the PKM2 expression through interaction with miR-7-5p, conferring resistance to L-OHP (Chen et al. 2024b). The circular RNA circRS-122 (hsa_circ_005963) up-regulates PKM2 expression by suppressing miR-122, thereby conferring L-OHP resistance. Interestingly, L-OHP-resistant cells can transfer circRS-122 to sensitive cells via exosomes, leading to the transmission of chemoresistance (Wang et al. 2020), which presents a significant challenge for CRC therapy.

Notably, cancer cells exhibiting drug resistance are capable of isolating exosomal PKM2, thereby facilitating the transmission of chemotherapy resistance. Wang et al. (2021c) indicated that in lung cancer, hypoxia induces DDP-resistant cells to secrete abundant exosomal PKM2, promoting glycolysis and the production of reductive metabolites that may counteract the effects of DDP-induced ROS. Furthermore, the metabolism reprogramming of cancer-associated fibroblasts (CAFs) mediated by exosomal PKM2 results in a shift towards glycolysis, creating an acidic microenvironment that ultimately confers and maintains DDP resistance. Similarly, hypoxia-induced TMZ-resistant glioma cells contain ample exosomal PKM2, which effectively transmits chemoresistance to sensitive glioma cells, further exacerbating the TMZ resistance of glioma (Li et al. 2024b).

Although the dysregulation of PKM2 is significantly associated with cancer chemoresistance, further efforts are needed to develop small molecular inhibitors or strategies to reverse the chemoresistance induced by PKM2, which continues to pose a challenge.

### LDHA

Lactate dehydrogenase, which includes LDHA and LDHB, is responsible for the reduction of pyruvate to lactate, thereby facilitating the process of glycolysis. The overexpression of LDHA has been demonstrated in multiple cancer types and is closely associated with cancer chemoresistance, which is summarized in Table 2 (Sharma et al. 2022; Feng et al. 2018; Li et al. 2022c).

In breast cancer, aberrant expression of LDHA-mediated glycolysis is correlated with the acquisition of TAM resistance in breast cancer (Papulino et al. 2024). Mechanistically, Das et al. (2019) substantiated that the overexpression of LDHA induces TAM resistance by interacting with Beclin-1. Moreover, LDHA-induced TAM resistance can be activated by the downregulation of Forkhead box O3 (FoxO3) (Fiorillo et al. 2023). In HCC, a factor that binds to the inducer of short transcripts-1 (FBI-1) upregulates LDHA expression through the stimulation of the hypoxia-inducible factor-1 alpha (HIF-1α) pathway by inhibiting miR-3692-5p, ultimately conferring resistance to molecular targeted agents in HCC cells, including sorafenib, regorafenib, lenvatinib, and cabozantinib (Liu et al. 2019). Acylphosphatase 1 (ACYP1) stabilizes the c-Myc protein through interaction with HSP90, leading to the up-regulation of LDHA. Therefore, the ACYP1/MYC/LDHA axis promotes glycolysis and confers lenvatinib resistance in HCC (Wang et al. 2023). GEM resistance commonly occurs in pancreatic cancer, potentially due to the upregulation of LDHA mediated by the family with sequence similarity 83 (Fam83) (Hua et al. 2021). Furthermore, LDHA expression has been shown to be stimulated by the nuclear glucose-regulated protein (GRP78)-HIF-1α complex under glucose deprivation, thereby conferring GEM resistance in pancreatic cancer (Zhao et al. 2023).

Additionally, post-transcriptional modifications and PTMs of LDHA regulate glycolysis and drug resistance. Methyltransferase 3 (METTL3) and insulin-like growth factor 2 mRNA-binding protein 2 (IGF2BP2) mediate m6 A modification by activating the transcription and translation of LDHA, which increases LDHA-mediated chemoresistance in CRC (Zhang et al. 2022; Jiang et al. 2022b). Regarding PTMs of the protein, Chen et al. (2024c) found that zinc finger DHHC-type containing 9 (ZDHH9) facilitates the palmitoylation of cysteine at site 163 of LDHA, enhancing its activity and conferring GEM resistance in pancreatic cancer cells.

The role of ncRNAs extends beyond the mediation of glycolysis through the direct regulation of glycolytic factors; it has also been demonstrated that they influence LDHA-mediated glycolysis via the establishment of a robust ceRNA network, which has been associated with the development of chemoresistance. Li et al. (2021) demonstrated that the upregulation of miR-329-3p suppresses glycolysis by targeting LDHA, thereby overcoming DDP resistance in osteosarcoma. In breast cancer, lncRNA DIO3OS (DIO3 Opposite Strand Upstream RNA) contributes to aromatase inhibitor resistance by enhancing the stability and expression of LDHA mRNA through PTBP1 (Chen et al. 2022b). Similarly, in gastric cancer, PTBP1-mediated upregulation of LDHA is also facilitated by the lncRNA SNHG16/miR-506-3p axis, which is linked to 5-FU resistance (Ding et al. 2022). Additionally, several other lncRNAs function as miRNA sponges and contribute to chemoresistance in gastric cancer. For instance, the lncRNA HAGLR/miR-338-3p (Hu et al. 2022b), lncRNA SNHG7/miR-34a (Pei et al. 2021), lncRNA DLEU2/miR-30c-5p (Xiang et al. 2024), and lncRNA NEAT1/miR-34a (Shao et al. 2021) axis have all been shown to upregulate LDHA expression, mediating resistance to 5-FU, DDP, and PTX, respectively. Regarding circRNAs, circUBE2D2 (hsa_circ_0005728) enhances LDHA expression by sponging miR-8893p, thereby conferring sorafenib resistance in HCC cells (Huang et al. 2021). Meanwhile, the circHIF1 A/miR-361-5p axis promotes LDHA expression by upregulating HIF-1α, subsequently inducing cetuximab resistance in CRC (Geng et al. 2024).

The intricate regulatory network of tumors encompasses an inextricable relationship among various factors and signaling pathways. Typically, the glucose metabolism reprogramming that induces chemoresistance is not limited to a single glycolytic enzyme. In fact, alterations in the expression of multiple glycolytic enzymes simultaneously impact cancer resistance to PTX, sorafenib, ADR, and DDP in breast cancer, HCC, AML, and CRC (Li et al. 2018; Liu et al. 2019; Liu et al. 2023; Ryu et al. 2019; Chen et al. 2024 d; Shi et al. 2021). Therefore, targeting multiple glycolytic genes concurrently may provide a novel approach to overcoming chemoresistance.

## Lactylation

**Fig. 3:**
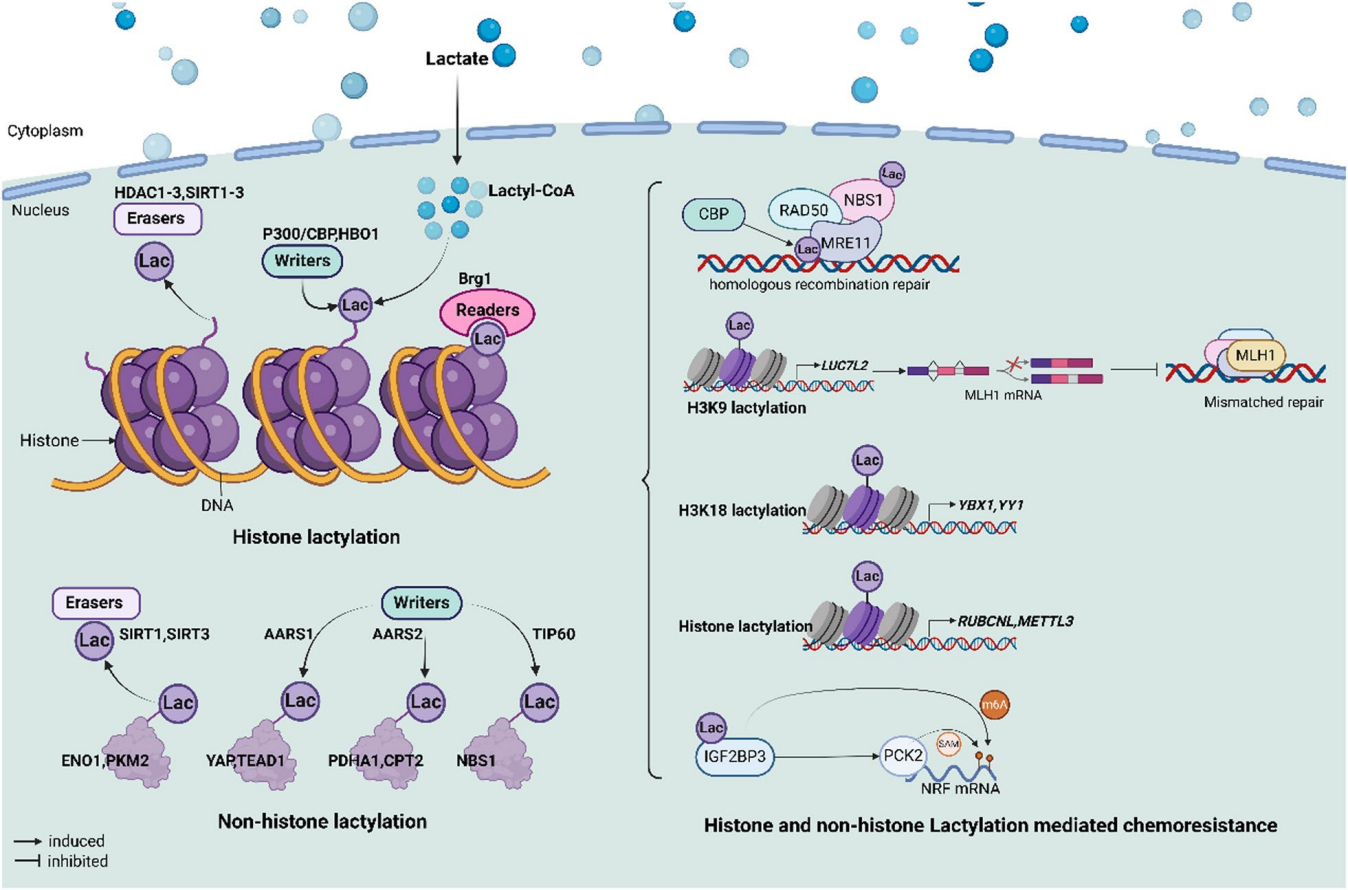
The core processes of histone and non-histone lactylation, along with the mechanisms underlying lactylation modifications in chemoresistance. Certain proteins function as “writers,” “readers,” and “erasers” in the context of histone and non-histone lactylation modification. Extensive lactylation modifications in tumor cells mediate chemoresistance by driving the expression of various genes and enhancing protein interactions, thereby activating downstream biological pathways

Abundant lactate, derived from high glycolytic flux, forms lactylation modifications (Kla) through covalent binding to lysine residues of proteins. When lactylation occurs on histones, referred to as histone lactylation, this process regulates the accessibility of DNA molecules and subsequently modulates gene expression (Jing et al. 2025). Additionally, lactylation is prevalent in non-histone proteins and plays a role in various biological processes by influencing protein function. Similar to other RNA and protein modifications, lactylation involves specific “writers”, “erasers”, and “readers” (He et al. 2024) (Fig. 3).

In the context of PTM, P300/CBP exhibits acetyltransferase activity and typically functions as an auxiliary transcriptional activator (Chen et al. 2022c). In recent years, P300/CBP has been identified as a “writer” of lactylation, mediating lactylation and promoting tumor progression (Yang et al. 2024; Yang et al. 2022). Recent studies have validated several novel lactylation “writers”: alanyl-tRNA synthetase (AARS1-2), which moonlights as a lactyl-transferase, TIP60 (also known as KAT5), HBO1 (also known as KAT7), and KAT8. In cancers, the intracellular accumulation of lactate leads to the import of AARS1 into the nucleus, where it lactylates the K90 site of YAP and the K108 site of TEA Domain Transcription Factor 1 (TEAD1) (Ju et al. 2024). Hypoxia induces lactylation of pyruvate dehydrogenase E1 subunit alpha 1 (PDHA1) and carnitine palmitoyl transferase 2 (CPT2) by AARS2 (Mao et al. 2024). Furthermore, TIP60 shares a similar structure with the acetyl-CoA-TIP60 complex, indicating that TIP60 functions as the “writer” of Nijmegen breakage syndrome (NBS1) lactylation at K388 (Chen et al. 2024e). HBO1 catalyzes the lactylation of histone H3 K9 to regulate gene transcription (Niu et al. 2024), while KAT8 “writes” lactyl-CoA to mediate the lactylation of elongation factor 1 alpha (eEF1 A) (Xie et al. 2024).

Two principal families of lysine deacetylases, including histone deacetylases (HDAC1-3) and Sirtuins (SIRT1-3), act as the “erasers” of lactylation. Moreno-Yruela et al. (2022) demonstrated that HDAC1-3 functions as delactylases and are effectively responsible for inhibiting overall histone lactylation, as well as the L-lactylation levels of H3 K18 and H4 K5. For SIRTs, Jennings et al. (2021) proved that SIRT2 regulates the lactylation of proteins. Recently, Du et al. (2024) reported that SIRT1 and SIRT3 act as powerful “erasers” of lysine residue lactylation (Kla), with SIRT1 specifically mediating a total of 1348 Kla sites and SIRT3 mediating 1143 Kla sites. Furthermore, they found that SIRT1 and SIRT3 target ENO1 at K228 la, and SIRT1 lactylates PKM2 at K207 la, inhibiting glycolysis and cell growth.

Research on histone lactylation ‘readers’ remains limited. Hu et al. (2024) found that Brg1 contains a bromodomain (BD) that acts as a reader of lactylation and co-localizes with lactylation of histone H3 K18 (H3 K18 la). Therefore, the discovery and study of lactylation provide significantly new insights into cancer research, particularly regarding chemoresistance.

## Lactylation and cancer chemoresistance

The occurrence of lactylation activates and modulates downstream factors that subsequently mediate cancer chemoresistance (see Fig. 3 and Table 3).

**Table 3:** Lactylations mediated cancer chemoresistance

Lactylation position	Downstream target	Drug resistance	Cancer	References
Histone of METTL3		ATRA	APL	Cheng et al. (2024)
Histone of RUBCNL	RUBCNL/BECN1 complex	Bevacizumab	CRC	Li et al. (2024c)
IGF2BP3	Stability of PCK and NRF2	Levatinib	HCC	Lu et al. (2024)
H3 K9	LUC7L2/MLH1/DNA mismatch repair	TMZ	GBM	Yue et al. (2024)
K673 of MRE11	DNA homologous recombination repair	DDP, Olaparib, ETOP	Pan-cancer	Chen et al. (2024f)
K338 of NBS11	DNA homologous recombination repair	DDP	Gastric cancer	Chen et al. (2024e)

Cheng et al. (2024) demonstrated that lactylation regulates METTL3, conferring resistance to all-trans retinoic acid (ATRA) in acute promyelocytic leukaemia (APL). In CRC, the enrichment of lactate in the tumor microenvironment mediates histone lactylation that facilitates the transcription of rubicon-like autophagy enhancer (RUBCNL). RUBCNL further promotes autophagosome maturation by recruiting the class III phosphatidylinositol 3-kinase complex through interaction with beclin 1 (BECN1), ultimately conferring resistance to resistance (Li et al. 2024c). Lactylation enhances the stability of mRNA and proteins. In HCC, Lu et al. (2024) demonstrated that the lactylation of IGF2BP3 stabilizes phosphorenolpyruvate carboxykinase (PCK) and nuclear factor erythroid 2-related factor 2 (NRF2), ultimately promoting resistance to levatinib.

Chemoresistance is closely related to DNA damage and repair; interestingly, lactylation has emerged as a novel contributing factor. In GEM cells, lactylation mediated DNA mismatch repair is crucial for the development of TMZ resistance. The lactylation of H3 K9 (H3 K9 la) enhances the expression of LUC7-like 2, a pre-mRNA splicing factor (LUC7L2), by activating its transcription, thereby inhibiting the mismatch repair of mutL homolog 1 (MLH1). Notably, GBM cells exhibit renewed sensitivity to the effects of TMZ when the activity of LDHA/LDHB is inhibited (Yue et al. 2024). Resistance to DDP, olaparib, and ETOP can partly be attributed to the repair of DNA double-strand breaks. Chen et al. (2024f) proposed that this may be due to MRE11 the K673 site lactylation by CREB-binding protein (CBP) acetyltransferase, which further enhances DNA-binding ability and end resection. Another potential mechanism involves LDHA-mediated hyperlactate inducing TIP60 to ‘write’ lactylation at the K338 site of NBS1, promoting the binding of the RAD50/NBS1/MRE11 complex to the sites of DNA double-strand breaks (Chen et al. 2024e).

The discovery of lactylation occurred just five years ago, and research into its role in cancer chemoresistance is still in its early stages. Nevertheless, existing studies suggest that lactylation plays a pivotal role in chemotherapy resistance, presenting a potential strategy for cancer treatment.

## Glycolysis and lactylation in chemoresistance: the synergistic effect

Glycolysis is inextricably linked to lactylation modifications. It provides a substrate for proteins to undergo lactylation by producing lactate. In turn, lactylation affects tumor cell metabolism and gene expression by altering protein function. This relationship plays a critical role in cancer chemoresistance.

**Fig. 4:**
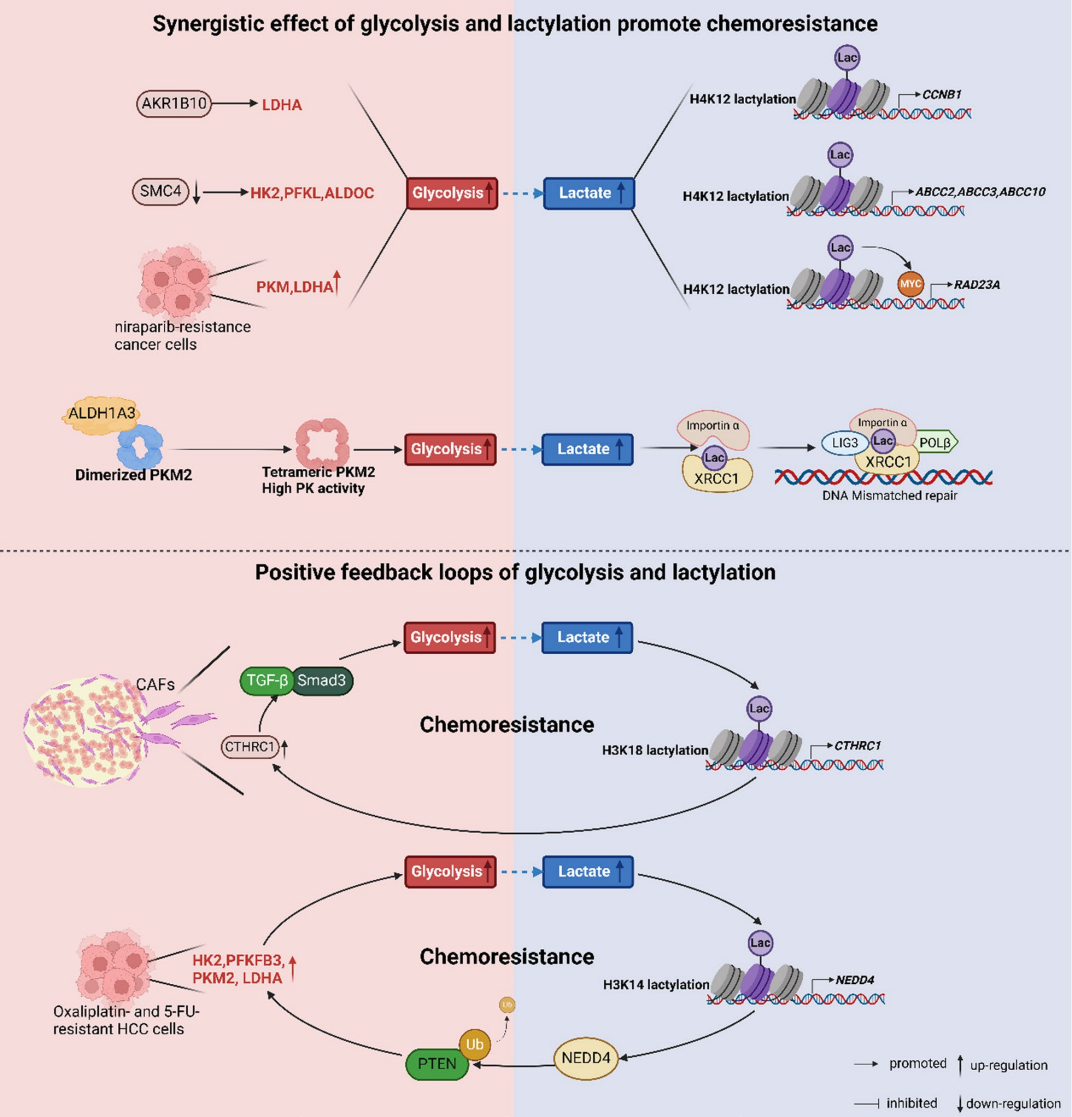
The synergistic effects of glycolysis and lactylation under cancer chemoresistance. In tumor cells or tumor-associated cells that develop chemotherapy resistance, the expression of oncogenic or oncostatic factors and the activation of aberrant signaling pathways promote glycolysis, leading to the accumulation of lactate. This accumulation subsequently mediates the lactylation modification of proteins. The alteration of lactylation levels influences protein functions and modulates downstream biological pathways, or sustains the activation of the glycolysis pathway, ultimately inducing chemoresistance

Recent studies have increasingly recognized that modifications in glycolysis and lactylation synergistically promote chemoresistance (see Fig. 4 and Table 4). Li et al. (2024 d) discovered that the metabolism of DDP-resistant cells is characterized by a significant enhancement of glycolysis and a down-regulation of the tricarboxylic acid cycle. This metabolic shift leads to the accumulation of lactate, which subsequently increases lactate levels and mediates the occurrence of lactylation. They demonstrated that the H3 K18 la in the promoter regions activates the transcription factors Y-box binding protein 1 (YBX1) and Yin Yang 1 (YY1), which induce DDP resistance in bladder cancer. Lung cancer-derived brain metastases exhibit resistance to pemetrexed, with both glycolysis and lactylation playing key roles. Mechanistically, Aldo–keto reductase family 1 B10 (AKR1B10) elevates glycolytic levels by increasing LDHA expression, leading to the accumulation of lactate, which activates the transcription of cyclin B1 (CCNB1) through the lactylation of histone H4 K12 (H4 K12 la) (Duan et al. 2023). Sun et al. (2023c) found that the attenuation of structural maintenance of chromosome 4 (SMC4) promotes glycolysis by upregulating HK2, phosphofructokinase liver type (PFKL), and ALDOC, subsequently enhancing lactate production. This process activates the H4 K12 la of ATP-binding cassette transporters (ABCC2, ABCC3, ABCC10), leading to increased drug efflux and conferring irinotecan resistance in diapause-like CRC cells.

**Table 4:** The synergistic effect of glycolysis and lactylation in cancer chemoresistance

Gene	Upregulation of glycolytic enzyme	Downstream lactylation targets	Drug resistance	Cancer	References
		H3 K18 of YBX1 and YY1	DDP	Bladder cancer	Li et al. (2024 d)
AKR1B10	LDHA	H4 K12 of CCNB1	Pemetrexed	Lung cancer-derived brain metastasis	Duan et al. (2023)
SMC4	HK2, PFKL, ALDOC	H4 K12 of ABCC2, ABCC3 and ABCC10	Irinotecan	CRC	Sun et al. (2023c)
ALDH1 A3	PKM2 tetramerization	XRCC1 K427		GBM	Li et al. (2024e)
	PKM, LDHA	H4 K12 of RAD23 A	Niraparib	Ovarian cancer	Lu et al. (2025)
	ALDOB	CEACAM6	5-FU	CRC	Chu et al. (2023)
CTHRC1		H3 K18 of CTHRC1	TKIs	Lung cancer	Zhang et al. (2025)
Ubiquitination of PTEN	HK2, PFKFB3, PKM2, LDHA	H3 K14 of NEDD4	Multidrug	HCC	Zeng et al. (2025)

Existing studies have confirmed that the lactylation of DNA damage repair-related proteins induced by elevated glycolysis promotes chemoresistance in cancer. Li et al. (2024e) demonstrated that aldehyde dehydrogenase 1 family member A3 (ALDH1 A3) interacts with PKM2, enhancing its tetramerization. The tetrameric form of PKM2, characterized by high glycolytic enzyme activity, promotes glycolysis and leads to the accumulation of intracellular lactate. This accumulation subsequently mediates the lactylation of XRCC1 at lysine 427 (K427), inducing DNA mismatch repair and ultimately conferring chemoresistance. Their research indicates that this mechanism also contributes to resistance against radiotherapy. Moreover, in niraparib-resistant ovarian cancer cells, the upregulation of PKM and LDHA expression enhances glycolysis, leading to lactic acid accumulation that induces H4 K12 la of RAD23 A. Notably, H4 K12 la enhances the recruitment of MYC to the RAD23 A promoter, further activating RAD23 A transcription and expression. Ultimately, this process promotes resistance to niraparib in ovarian cancer by enhancing DNA damage repair (Lu et al. 2025).

Additionally, lactate secretion mediates lactylation in neighboring cells, which also promotes and maintains chemoresistance. Chu et al. (2023) demonstrated that aberrant expression of ALDOB enhances glycolysis and increases lactate secretion, subsequently stimulating the lactylation and stabilization of the cell adhesion molecule carcinoembryonic antigen-related cell adhesion molecule 6 (CEACAM6) in neighboring CRC cells. When bioenergetics are altered through the application of glycolytic pathway inhibitors, CEACAM6 levels are significantly reduced. This finding suggests that the increased glycolytic capacity and the occurrence of lactylation are primary contributors to elevated CEACAM6 levels, serving as a key factor in conferring and transmitting resistance to 5-FU in CRC.

It is noteworthy that glycolysis and histone lactylation can create a positive feedback loop that fosters chemotherapy resistance. Zhang et al. (2025) demonstrated that in EGFR-TKI-resistant lung cancer, cancer-associated fibroblasts (CAFs) overexpressing collagen triple helix repeat-containing 1 (CTHRC1) activated TGF-β/Smad3 signaling to enhance glycolysis in cancer cells. The substantial lactate produced during glycolysis induced H3 K18 la in CAFs, further upregulating CTHRC1 expression. This process ultimately established a positive feedback loop that sustains lung cancer resistance to EGFR-TKI. Additionally, in oxaliplatin- and 5-FU-resistant HCC cells, elevated levels of HK2, PFKFB3, PKM2, and LDHA enhance glycolysis, leading to substantial lactate production. The accumulation of lactate mediates histone H3 K14 lactylation (H3 K14 la), thereby activating the transcription of NEDD4, which facilitates the ubiquitylation of the target protein PTEN. The down-regulation of PTEN subsequently affects glycolytic enzymes, further promoting glycolysis. Thus, a positive feedback loop involving glycolysis, H3 K14 la, NEDD4, and PTEN contributes to multidrug resistance in HCC (Zeng et al. 2025).

These studies have elucidated the close connection between glycolysis and lactylation in cancer chemotherapy resistance, further refining the network of relationships between tumor metabolic reprogramming and epigenetic regulation. This connection provides a more comprehensive perspective for understanding the occurrence mechanisms underlying chemoresistance and presents new strategies for overcoming it.

## Inhibitors overcome cancer drug resistance

In the histone lactylation first reported by Zhao’s team, the application of the PDH and LHDA inhibitors sodium dichloroacetate (DCA) and Oxamate significantly inhibited lactate production and histone lactylation (Zhang et al. 2019a). This suggests that the application of glycolysis inhibitors may also regulate the level of protein lactylation by inhibiting glycolysis. Given the important regulatory role of glycolysis and its induced protein lactylation in cancer chemoresistance, targeting glycolysis or targeting histone and non-histone lactylation represents a more promising therapeutic strategy to overcome chemoresistance.

**Fig. 5:**
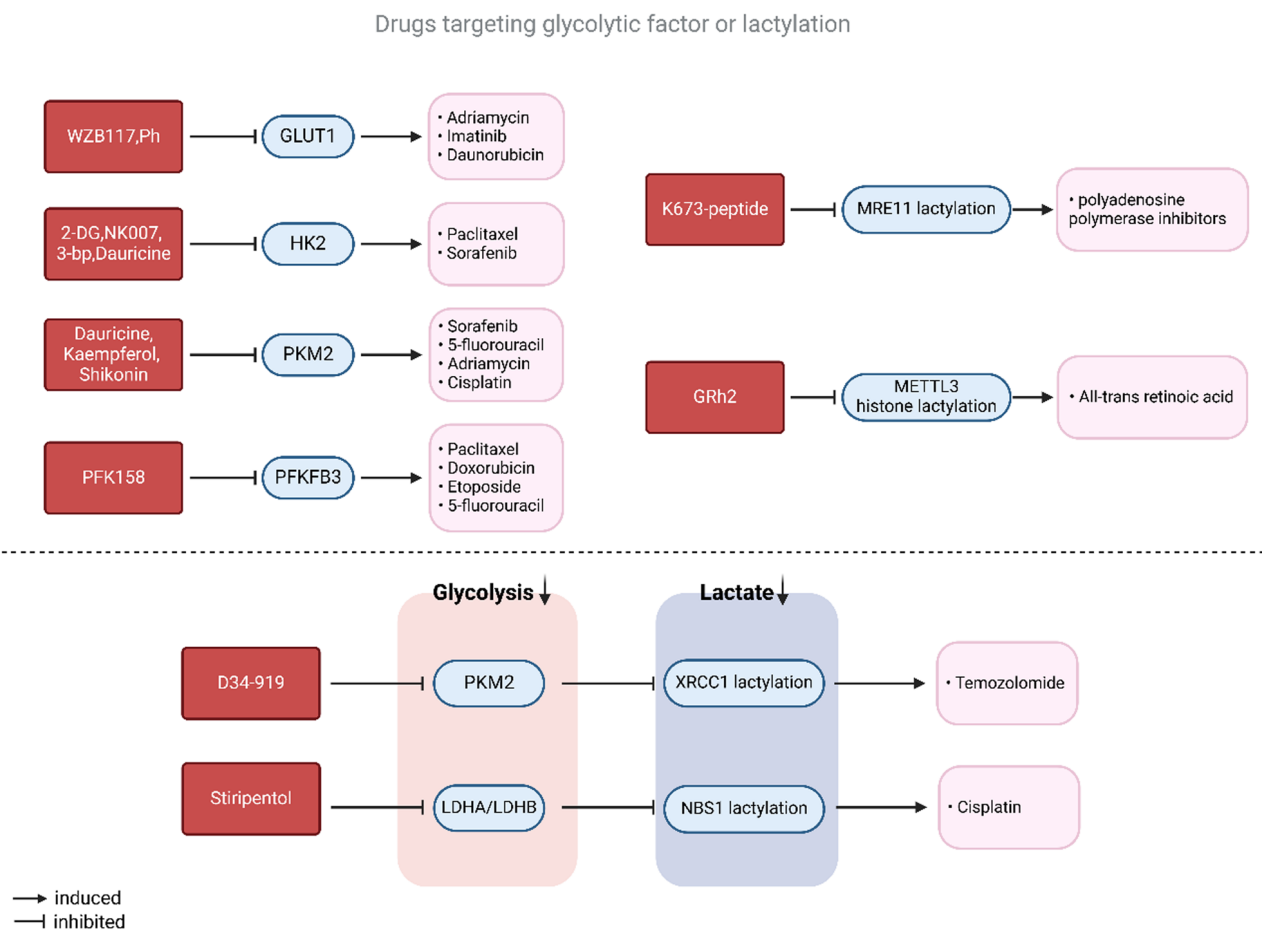
Inhibitors overcome cancer chemoresistance. Inhibitors targeting glycolytic enzymes or lactylation that are capable of reversing chemotherapy resistance, as reported in existing research, could be a potential and desirable therapeutic strategy to overcome tumor resistance

The use of inhibitors targeting glycolytic enzymes, either alone or in combination with other chemotherapeutic agents, has been demonstrated to effectively overcome chemotherapy resistance in cancer (Fig. 5 and Table 5). As a specific GLUT1 inhibitor, WZB117 not only resensitizes resistant breast cancer cells to ADR (Chen et al. 2017), but also effectively overcomes resistance in gastrointestinal stromal tumor cells when combined with imatinib (Shima et al. 2022). The polyphenol phloretin (Ph), isolated from apples, significantly antagonizes GLUT1 in daunorubicin-resistant breast cancer cells and CRC cells, markedly inhibiting glucose uptake and inducing apoptosis in daunorubicin-resistant cells, thereby enhancing chemotherapeutic efficacy (Cao et al. 2007).

**Table 5:** Inhibitors overcoming chemoresistance

Inhibitors	Target	Effects in chemoresistance	Cancer	References
WZB117	GLUT1	Overcome ADR resistance	Breast cancer	Chen et al. (2017)
		Combined with imatinib rescue chemoresistance	Gastrointestinal stromal tumor	Shima et al. (2022)
Phloretin	Antagonize GLUT1	Overcome daunorubicin resistance	Breast cancer, CRC	Songyang et al. (2022)
2-DG	HK	Combined with Bcl-2 antagonists enhance the drug effect	Prostatic cancer	Yamaguchi et al. (2011)
NK007	HK2	Sensitization to PTX	Ovarian cancer	Li et al. (2019)
3-bp	HK2	Reverse L-OHP resistance; Used with L-OHP inhibits cancer progression	CRC	Zhang et al. (2021)
Dauricine	HK2, PKM2	Sensitization to sorafenib	HCC	Li et al. (2018)
Kaempferol	PKM2	Overcome 5-FU resistance	CRC	Wu et al. (2022)
Shikonin	PKM2	Enhances DDP effect	Bladder cancer	Wang et al. (2018); Wang et al. (2017)
		Sensitization to ADR and DDP	HCC	Martin et al. (2020)
PFK158	PFKFB3	Reverse PTX resistance	Ovarian cancer, cervical cancer	Mondal et al. (2019)
		Enhanced effects of DOX, ETOP and 5-FU	Small-cell lung carcinoma	Lypova et al. (2019)
GRh2	Lactylation of METTL3	Sensitization to ATRA	APL	Cheng et al. (2024)
K673-peptide	Lactylation of MRE11	Overcome PARPi resistance	CRC	Chen et al. (2024f)
D34-919	Interaction of PKM2 and ALDH1 A3	Overcome TMZ resistance	GBM	Li et al. (2024e)
Stiripentol	LDHA/LDHB	Reverse TMZ resistance; Combined with DDP enhances drug effect	GBM	Yue et al. (2024)

The well-known glycolysis inhibitor 2-deoxy-D-glucose (2-DG) effectively inhibits glycolysis by targeting hexokinase (HK). Yamaguchi et al. (2011) demonstrated that 2-DG, when combined with two Bcl-2 antagonists, ABT-263 and ABT-737, exhibited significant efficacy in xenograft mouse models of highly metastatic, chemoresistance human prostate cancer cells. While 2-DG has been regarded as having strong potential for treating solid tumors and hormone-refractory prostate cancer, clinical trials were ultimately terminated after reaching phase I or II due to its failure to significantly inhibit tumor growth in vivo (NCT00633087). Li et al. (2019) demonstrated that NK007, a (±)-tylophorine malate isolated from the Asclepiadaceae family, significantly resensitizes ovarian cancer cells to PTX by inducing the degradation of HK2. Additionally, 3-bromopyruvate (3-bp), another HK2 inhibitor, has been shown to reverse L-OHP resistance in CRC, and the combination of 3-bp and L-OHP effectively suppresses tumor progression compared to L-OHP treatment alone (Zhang et al. 2021). Dauricine, a natural alkaloid, inhibits HK2 and PKM2 by increasing miR-199a, thereby rendering HCC cells more susceptible to sorafenib (Li et al. 2018). Furthermore, Wu et al. (2022) found that Kaempferol reverses 5-FU resistance in CRC cells through the miR-326-hnRNPA1/A2/PTBP1-PKM2 axis. Shikonin, a naphthoquinone, significantly inhibits the activity of PKM2 and induces necrotic apoptosis in drug-resistant cells. The application of Shikonin enhances the efficacy of DDP in the treatment of bladder cancer, with the combination therapy demonstrating a more pronounced effect (Wang et al. 2018; Wang et al. 2017). In HCC, it is well established that conventional systemic chemotherapy is largely ineffective (Zhu 2006), while transarterial embolisation chemotherapy (TACE) is the preferred procedure for patients with intermediate-stage HCC. However, over 40% of patients do not respond to TACE, making the overcoming of drug resistance a significant challenge in HCC treatment. TACE is most frequently combined with single-agent chemotherapy, with ADR and DDP being the two most commonly used agents. Martin et al. (2020) demonstrated that the inhibition of PKM2 by Shikonin could enhance the sensitivity of HCC cells to ADR and DDP, with the combination of Shikonin and ADR showing a more significant effect.

The activity and expression of PFKFB3 are inhibited by the novel inhibitor PFK158. In ovarian and cervical cancers, PFK158 effectively reverses PTX resistance by suppressing PFKFB3-related glycolysis and inducing apoptosis (Mondal et al. 2019). In small cell lung carcinoma, the application of PFK158 can enhance the anti-tumor effects of ABCG2-targeting drugs such as DOX, etoposide, and 5-FU in conditions enriched with cancer stem cells (Lypova et al. 2019). In CRC, the combined use of PFK158 and L-OHP significantly inhibits tumor progression and prevents the development of chemoresistance (Yan et al. 2019). Furthermore, in multiple advanced drug-resistant breast cancer models, the integrated application of PFKFB3 inhibitors and ER+ targeted therapies demonstrate high efficacy in overcoming chemoresistance, including TAM and PTX (Truong et al. 2021). Notably, PFK158 is the first inhibitor targeting PFKFB3 to enter phase I clinical trials (NCT02044861), and it may hold significant clinical utility as a monotherapy or in combination with targeted agents (Redman et al. 2015).

In addition, targeting histone or non-histone lactate modification processes is a potential strategy to overcome chemoresistance. The compound 20(S)-ginsenoside Rh2 (GRh2) has been reported as a histone deacetylase inhibitor and has been shown to enhance the sensitivity of all-trans retinoic acid (ATRA) in ATRA-resistant acute promyelocytic leukemia (APL) cells, exhibiting effects similar to those of histone lactylation inhibitors (Cheng et al. 2024).

K673-peptide-3 (K673-pe), designed for the MRE11 K673 locus, effectively inhibits MRE11 non-histone lactylation, thereby disrupting the DNA homologous recombination repair and overcoming sensitivity to chemoresistance in colorectal cancer and polyadenosine polymerase inhibitors (PARPi) (Chen et al. 2024f). D34-919, a small molecule compound identified by Li et al., blocks the interaction of ALDH1 A3 with PKM2, inhibits downstream XRCC1 non-histone lactylation, and further impedes DNA damage repair. This mechanism helps overcome resistance to temozolomide in glioma-like organ models while enhancing sensitivity to radiotherapy (Li et al. 2024e). The LDHA/LDHB inhibitor Stiripentol disrupts lactate production and lactylation, thereby interfering with the DNA repair mechanism, and has demonstrated efficacy in overcoming TMZ resistance in GBMs. Moreover, Stiripentol acts as a chemosensitizing agent in combination with DDP, enhancing the cytotoxicity of DDP and overcoming chemoresistance. The safety of Stiripentol, a clinically used drug for treating childhood epilepsy, is well established, and its potential as a chemotherapeutic sensitizing agent targeting protein lactylation shows promise for clinical translation and therapy (Yue et al. 2024).

These studies have demonstrated that glycolytic enzymes and lactylation are promising targets in cancer therapy. Furthermore, the recent discovery regarding Stiripentol strongly suggests that co-targeting glycolytic enzymes and lactylation may represent more feasible and effective therapeutic strategies.

## Discussion

The significance of glycolytic reprogramming in tumors related to cancer chemoresistance has been well established. This phenomenon is closely linked to the activation and regulation of key glycolytic factors by oncogenic signals and non-coding RNAs, as well as the modification of these factors at both the transcriptional and translational levels. The application of targeted glycolytic enzyme inhibitors has demonstrated the potential to overcome chemotherapy resistance and exhibit significant anti-tumor activity. Specifically, GEN-27 and lonidamine inhibit tumor progression by targeting HK2 (Tao et al. 2017), while Benserazide inhibit tumor progression by targeting PKM2 (Li et al. 2017b). Although these inhibitors have not been extensively reported in relation to chemoresistance, they nonetheless demonstrate the feasibility of targeting glycolysis. In addition to their classic enzymatic functions, the non-enzymatic roles of glycolytic factors warrant consideration. For instance, ENO1 is involved in the regulation of phospholipid metabolism through its non-enzymatic function (Ma et al. 2023), thereby bridging glycolysis and other metabolic pathways. Furthermore, the role of exosomes in mediating crosstalk between glycolytic enzymes, such as PKM2, and the tumor microenvironment presents an important area for discussion and exploration, as this interaction may enable chemosensitive cells to develop resistance to chemotherapy (Li et al. 2024b). These findings suggest that the metabolic regulatory mechanisms in cancer cells are complex and diverse, involving multiple signaling pathways and molecular mechanisms, and that single-targeted therapies may not represent the most optimal and effective strategy to overcome chemoresistance.

Lactate is the most significant by-product of glycolysis (Sun et al. 2017) and serves as a substrate for lactylation. Notably, a positive feedback loop regulatory mechanism exists between glycolysis and lactylation. In PDAC, the H3 K18 lactylation upregulates the expression of TTK, which subsequently phosphorylates and activates LDHA, ultimately promoting glycolysis and lactylation. This accumulation, in turn, influences histone lactylation and facilitates tumor progression (Li et al. 2024f). Thus, it’s imperative to consider the interplay between glycolysis and lactylation in tumors rather than examining them in isolation. The positive feedback regulatory mechanisms of glycolysis and lactylation contribute significantly to cancer chemoresistance. This connection between metabolic reprogramming and epigenetic regulation offers new insights into cancer development and drug resistance, suggesting that co-targeting glycolysis and lactylation may represent a more effective oncological strategy. A prime example of such a co-targeting approach is the ‘new use of an old drug’, Stiripentol (Chen et al. 2024e). A comprehensive understanding and exploration of the relationship and regulatory mechanisms between glycolysis and lactylation are essential for a deeper insight into the complex regulatory networks at the metabolic and epigenetic levels of cancer, as well as for the development of effective targeted therapies, which still require further investigation.

However, there are numerous challenges in translating metabolic inhibitors into clinical practice. For instance, 2-DG was regarded as having significant potential for the treatment of solid tumors and hormone-refractory prostate cancer (Stein et al. 2010). Nevertheless, the trial was terminated after entering phase I or II clinical trials due to its failure to significantly inhibit tumor growth in vivo. Despite the notable antitumor activity exhibited by metabolic inhibitors in preclinical studies, tumor heterogeneity and the complexity of tumor metabolism present substantial obstacles to their clinical translation. Additionally, tumor metabolism exhibits plasticity, and the relationship between energy metabolism and immune function plays a crucial role in the immune escape of cancer cells. Targeting the glycolytic pathway and the PD-1/PD-L1 axis may enhance anti-tumor immune responses. (Liu et al. 2025). For instance, the small molecule activator of PKM2, TP-1454, has been the focus of a phase 1 clinical trial assessing the safety of once-daily oral TP-1454 monotherapy and its combination with the PD-1 inhibitor nivolumab in patients with advanced metastatic or progressive solid tumors (NCT04328740). Furthermore, glycolysis-induced lactylation modifications interact with other metabolic processes. For example, lactylation of IGF2BP3 ultimately drives the reprogramming of serine metabolism in HCC and ultimately contributes to Lenvatinib resistance (Lu et al. 2024). These factors indicate that the complexity, heterogeneity, and plasticity of tumor metabolism pose significant challenges for the clinical application of metabolic inhibitors. Therefore, exploring multi-targeted combination therapies or developing more selective metabolic inhibitors is essential for future research.

The close relationship between glycolysis, lactylation, and cancer chemoresistance reveals the complex regulatory mechanisms of tumor cells at both the metabolic and epigenetic levels. A deeper understanding of this relationship can provide an important theoretical basis and potential targets for the development of novel anticancer therapies. Future studies should further explore the specific molecular mechanisms of histone and non-histone lactyation, as well as the regulatory network of the positive feedback loop between glycolysis and lactylation. Additionally, the future investigations should focus on the development of relevant multi-target combination therapies and their potential applications in cancer therapy and chemoresistance. In conclusion, targeting glycolysis and lactylation remains a promising and innovative strategy for overcoming cancer chemoresistance and inhibiting tumor progression, despite the challenges facing the field.

## Data Availability

No datasets were generated or analysed during the current study.

## Abbreviations

PTM: Post-translational modification
ncRNA: Non-coding RNA
miRNA: Micro RNA
lncRNA: Long non-coding RNA
circRNA: Circular RNA
ceRNA: Competitive endogenous RNA
GLUT: Facilitative glucose transporter
HK: Hexokinase
PFKP: Phosphofructokinase, platelet
PFKFB3: 6-Phosphofructo-2-kinase/fructose-2,6-bisphosphatase 3
ALDO: Aldolase
PGAM1: Phosphoglycerate mutase 1
ENO1: Alpha-enolase
PKM: Pyruvate kinase
LDH: Lactate dehydrogenase
NFAT5: Nuclear factor of activated T cells 5
PTEN: Phosphate and tension homology deleted on chromosome ten
Zeb1: Zinc finger E-box binding homeobox 1
HER2: Human epidermal growth factor receptor-2
CSC: Cancer stem cells
PU.: Ets transcription factor
STAT3: Signal transducer and activator of transcription 3
PELP1: Proline, glutamic acid, leucine-rich protein 1
CHIP: C-terminus of Hsc70-interacting protein
SHP-1: Tyrosine phosphatase Src homology region 2 domain-containing phosphatase1
POU2 F1: POU domain 2-like transcription factor 1
MSI1: Musashi RNA binding protein 1
Tregs: Tumor-infiltrating regulatory T cells
PI3 K: Phosphatidylinositol 3-kinase
YAP1: Yes‐associated protein 1
PTBP1: Polypyrimidine tract-binding protein 1
hnRNP: Heterogeneous nuclear ribonucleoprotein
HIF-1α: Hypoxia-inducible factor-1 alpha
USP21: Ubiquitin-specific protease 21
PRMT6: Protein arginine methyltransferase 6
FBXW7: The F-Box and WD repeat domain containing 7
NAMPT: Nicotinamide phosphoribosyltransferase
KIF2 C: Kinesin family member 2C
PCAF: P300/CBP-associated factor
EC: Endometrial cancer
TKI: Tyrosine kinase inhibitors
CML: Chronic myeloid leukemia
TCF4: Transcription factor 4
JAK: Janus tyrosine Kinase
STAT: Signal transducer and activator of transcription
HOTAIR: Homeobox antisense intergenic RNA
PI3 K: Phosphatidylinositol 3-kinase
CEACAM6: Carcinoembryonic antigen-related cell adhesion molecule 6
ERK1/2: Extracellular regulated protein kinases 1/2
FBI-1: A factor that binds to inducer of short transcripts-1
ACYP1: Acylphosphatase 1
Fam83: Family with sequence similarity 83
GRP78: Nuclear glucose-regulated protein
METTL3: Methyltransferase 3
IGF2BP2/IGF2BP3: Insulin-like growth factor 2 mRNA-binding protein 2/3
m6 A: N6-methyladenosine
lncRNA DIO3OS: Long non-coding RNA DIO3 opposite strand upstream RNA
lncRNA SNHG7: Long non-coding RNA small nucleolar RNA host gene 7
lncRNA SNHG16: Long non-coding RNA small nucleolar RNA host gene 16
lncRNA HAGLR: Long non-coding RNA HOXD antisense growth-associated long non-coding RNA
lncRNA HOTAIR: Long non-coding RNA homeobox antisense intergenic RNA
lncRNA FLEU2: Long non-coding RNA deleted in lymphocytic leukemia 2
lncRNA H19: Long non-coding RNA H19 imprinted maternally expressed transcript
circUBE2D2: Hsa_circ_0005728
circHIF1 A: Circular RNA hypoxia-inducible factor 1 A
circHIF1 A: The CREB-binding protein
AARS1/AARS2: Alanyl-tRNA synthetase 1/2
PDHA1: Pyruvate dehydrogenase E1 subunit alpha 1
TEAD1: TEA Domain Transcription Factor 1
CPT2: Carnitine palmitoyltransferase 2
NBS1: Nijmegen breakage syndrome
eEF1 A: Elongation factor 1 alpha
HDAC: Histone deacetylases
SIRT: Sirtuins
Kla: Lysine residues lactylation
YBX1: Y-box binding protein 1
YY1: Yin Yang 1
AKR1B10: Aldo–keto reductase family 1 B10
CCNB1: Cyclin B1
RUBCNL: Rubicon-like autophagy enhancer
BECN1: Beclin 1
SMC4: Structural maintenance of chromosomes 4
PCK: Phosphoenolpyruvate carboxykinase
NRF2: Nuclear factor erythroid 2-related factor 2
CEACAM6: CEA cell adhesion molecule-6
ALDH1 A3: Aldehyde dehydrogenase 1 family member A3
LUC7L2: LUC7-like 2, pre-mRNA splicing factor
MLH1: MutL homolog 1
RAD50: RAD50 double strand break repair protein
MRE11: MRE11 homolog, double strand break repair nuclease
H4 K12 la: Lactylation of histone H4K12
H3 K9 la: Lactylation of H3K9
2-DG: 2-Deoxy-D-glucose
PET-CT: Positron emission tomography-computedtomography
CRC: Colorectal cancer
HCC: Hepatocellular carcinoma
NSCLC: Non-small cell lung cancer
SCLC: Small-cell lung carcinoma
TNBC: Triple-negative breast cancer
APL: Acute promyelocytic leukaemia
LSCC: Laryngeal squamous cell carcinoma
ER: Endoplasmic reticulum
ROS: Reactive oxygen species
GBM: Glioblastoma
AML: Acute myeloid leukemia
DOX: Doxorubicin
GEM: Gemcitabine
DDP: Cisplatin
5-FU: 5-Fluorouracil
PTX: Paclitaxel
L-OHP: Oxaliplatin
TMZ: Temozolomide
ADR: Adriamycin
EPI: Epirubicin
ETOP: Etoposide
TAM: Tamoxifen
Ara-C: Cytarabine
DTX: Docetaxel
D9D: Anti-CTLA-4 therapy
ATRA: All-trans retinoic acid
DCA: Sodium dichloroacetate
3-bp: 3-Bromopyruvate
GRh2: 20(S)-ginsenoside Rh2
